# Sex differences and individual variability in the captive Jamaican fruit bat (*Artibeus jamaicensis*) intestinal microbiome and metabolome

**DOI:** 10.1038/s41598-024-53645-5

**Published:** 2024-02-09

**Authors:** Jade C. Riopelle, Amirhossein Shamsaddini, Myndi G. Holbrook, Eric Bohrnsen, Yue Zhang, Jamie Lovaglio, Kathleen Cordova, Patrick Hanley, Lon V. Kendall, Catharine M. Bosio, Tony Schountz, Benjamin Schwarz, Vincent J. Munster, Julia R. Port

**Affiliations:** 1 Laboratory of Virology, Division of Intramural Research, National Institute of Allergy and Infectious Diseases, National Institutes of Health, Hamilton, MT USA; 2 Research Technologies Branch, Division of Intramural Research, National Institute of Allergy and Infectious Diseases, National Institutes of Health, Hamilton, MT USA; 3 Integrated Data Sciences Section, Research Technologies Branch, National Institute of Allergy and Infectious Diseases, National Institutes of Health, Bethesda, MD USA; 4 Rocky Mountain Veterinary Branch, Division of Intramural Research, National Institute of Allergy and Infectious Diseases, National Institutes of Health, Hamilton, MT USA; 5 Department of Microbiology, Immunology, and Pathology, Colorado State University, Fort Collins, CO USA; 6 Laboratory of Bacteriology, Division of Intramural Research, National Institute of Allergy and Infectious Diseases, National Institutes of Health, Hamilton, MT USA

**Keywords:** Microbiology, Microbial communities, Environmental microbiology

## Abstract

The intestinal microbiome plays an important role in mammalian health, disease, and immune function. In light of this function, recent studies have aimed to characterize the microbiomes of various bat species, which are noteworthy for their roles as reservoir hosts for several viruses known to be highly pathogenic in other mammals. Despite ongoing bat microbiome research, its role in immune function and disease, especially the effects of changes in the microbiome on host health, remains nebulous. Here, we describe a novel methodology to investigate the intestinal microbiome of captive Jamaican fruit bats (*Artibeus jamaicensis*). We observed a high degree of individual variation in addition to sex- and cohort-linked differences. The intestinal microbiome was correlated with intestinal metabolite composition, possibly contributing to differences in immune status. This work provides a basis for future infection and field studies to examine in detail the role of the intestinal microbiome in antiviral immunity.

## Introduction

Intestinal metabolism and microbiome composition are increasingly implicated in human health outcomes, especially disease course and severity of viral infections^1–6^. A growing body of literature indicates that the intestinal microbiome plays a functional role in many mammals, in part via the synthesis of metabolites that may influence intestinal immunity through responses to infection^6–10^. Among the most well-established of these associations is the production of various short chain fatty acids (SCFAs), which perform immunoregulatory functions, by bacterial species commonly found in human intestinal microbiomes^11–13^.

Bats are the reservoir hosts of viruses in several families, including Filoviridae, Paramyxoviridae, and Coronaviridae, that can cause severe disease in humans^14–17^. There are data indicating that bats, as the only flight-adapted mammals, have developed methods for reducing inflammation incurred during flight. These include reducing inflammatory cytokines such as TNF^18^, loss of the PYHIN gene family^19^, reduced activation of the NLRP3 inflammasome^20^, downregulation of caspase-1-mediated inflammasome activation^21^, dampened interferon activation as a result of STING mutation^22^, and increased expression of inflammasome-suppressing ASC2^23^. There is also evidence supporting the constitutive expression of innate immune genes, such as interferon-stimulated genes^24,25^, to reduce flight-induced oxidative metabolism and DNA damage^22,26^ in bats.

Flight has also been implicated in the unique signatures of bat intestinal microbiomes^27–29^. Furthermore, the intestinal microbiomes of several bat species are characterized by high abundances of *Pseudomonadota* and low abundances of *Bacteroidete*s, a suggested hallmark of dysbiosis in humans^30,31^. However, transplants of bat intestinal microbiomes to mice infected with H1N1 influenza virus lead to decreased inflammatory responses and increased survival rates^32^. Thus, microbial signatures that constitute dysregulation in other mammalian species may in fact contribute to bat immune responses to viral infections, potentially contributing to their posited immune tolerance^33,34^.

The Jamaican fruit bat (*Artibeus jamaicensis*) harbors diverse coronaviruses in the wild^35^ and has previously been used as a bat reservoir host model for MERS-CoV infection^36^. They are susceptible to a broad range of viruses after experimental inoculation, demonstrating Tacaribe virus and rabies virus infections of varying severity depending on inoculation dose and virus strain, respectively, asymptomatic Zika virus infection, and clinically mild bat H18N11 influenza A virus infection^37–40^. Despite their use as a reservoir host model and susceptibility to several highly pathogenic viruses, Jamaican fruit bats remain understudied with regards to their intestinal microbiome and immune response to infection, partially due to the difficulty of controlled studies under high containment conditions.

Here, we established novel methods for intestinal microbiome analysis of a captive colony of Jamaican fruit bats under conditions mimicking those of a prototypical infection study in high containment and characterized sex- and cohort-specific differences in the microbiome. We then conducted a metabolomic investigation in relation to differences observed in the intestinal microbiome. This work will provide a foundation for future infection studies to elucidate immune responses and mechanisms of immune tolerance of a model reservoir host species for newly emerging and re-emerging viruses.

## Results

### Microbiome analysis methods establishment for samples collected in maximum containment

We established novel methods to conduct microbiome analyses of samples obtained from animals under maximum containment (BSL-4) conditions, which can be applied to bats infected with BSL-3 and BSL-4 pathogens. Given the restrictions on sample removal and inactivation from high containment, the methods developed here incorporated acceptable inactivation techniques. We first established protocols for inactivating and extracting bacterial DNA from bat rectal swabs by testing three commercially available methods (Supplementary Fig. 1a,b): TRIzol Reagent (ThermoFisher), the AllPrep DNA/RNA Kit (Qiagen), and the QIAamp Fast DNA Stool Mini Kit (Qiagen). We determined that extraction with TRIzol reagent produced the highest yield of DNA.

Jamaican fruit bat fecal samples and rectal swabs were then compared after TRIzol extraction to establish downstream sampling methodology (Supplementary Fig. 1c). We tested both sample types with a PCR protocol for 16S ribosomal DNA based on manufacturer’s recommendations (Illumina) and modified accordingly^41^. In this study of Jamaican fruit bats—which produce soft, semi-formed fecal samples—rectal swabs both produced cleaner PCR products than did fecal samples and allowed for accurate longitudinal sampling of individuals.

### Microbial community composition of captive Jamaican fruit bats

**Figure 1:**
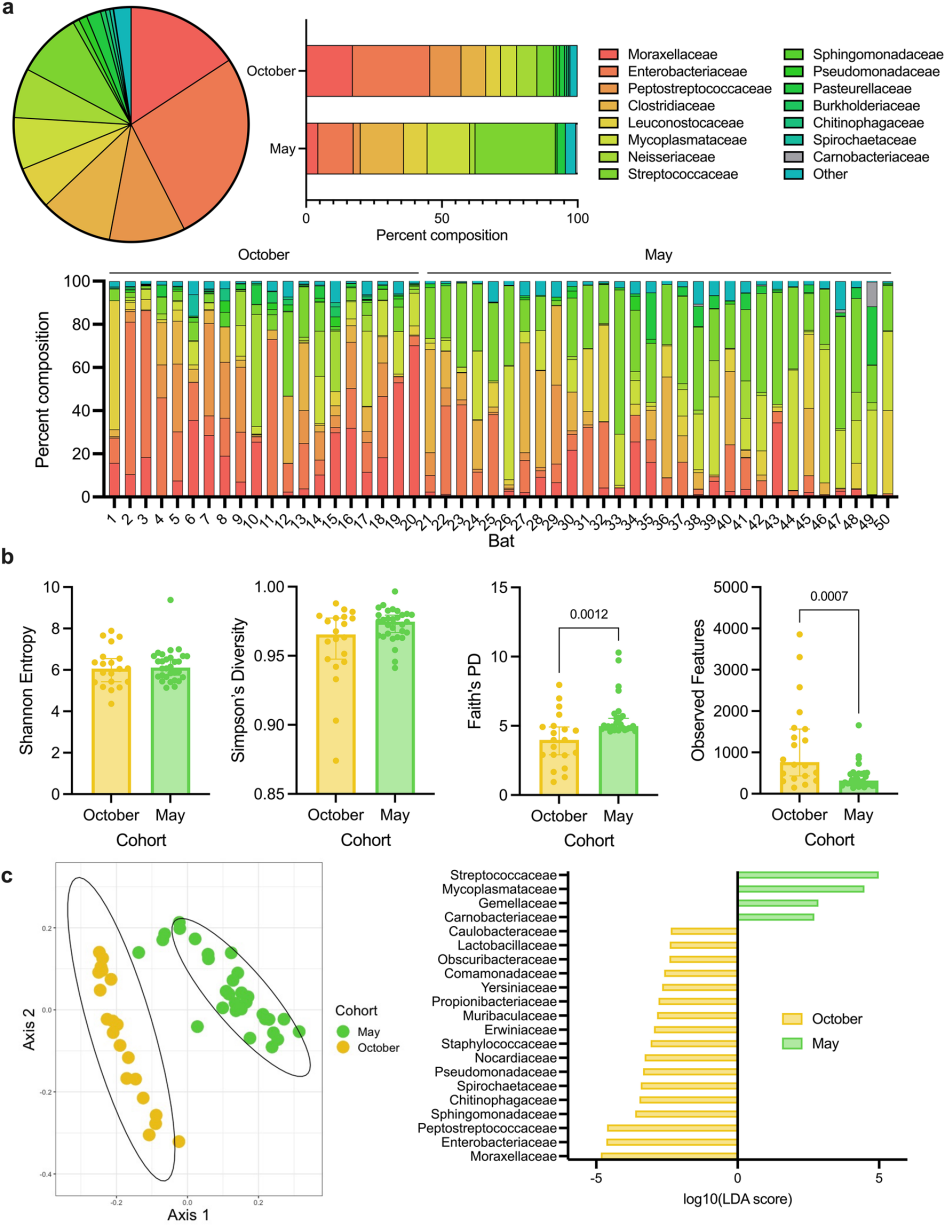
Differences between two adult cohorts (October, N = 20 and May, N = 30) of captive Jamaican fruit bats (*Artibeus jamaicensis*) in intestinal microbial community composition and diversity immediately after relocation between facilities. Samples taken after acclimation. (**a**) Percent abundance of selected microbial families in the intestinal tract across all bats (pie chart), by cohort (top right), and by individual bat (bottom). Unclassified reads at the family level and reads classified as mitochondria, chloroplasts, or non-prokaryotes were removed. Families with less than 0.5% abundance in either cohort were categorized as “Other” for visualization purposes. (**b**) Alpha diversity metrics (left to right: Shannon entropy, Simpson’s diversity, Faith’s phylogenetic diversity, and observed features) of the intestinal microbiome by cohort. Median and 95% confidence intervals with individual points overlaid. Significant p-values indicated; Mann–Whitney test (N = 20/30). (**c**) Principal coordinate analysis of unweighted UniFrac distance showing microbial community composition differences (left) and log_10_(linear discriminant analysis score) of differentially abundant families (LEfSE, p < 0.05) between cohorts (right).

We first analyzed the captive Jamaican fruit bat intestinal microbiome after acclimation of two cohorts shipped to our facility in October 2021 and May 2022 (N = 20 and N = 30, respectively), characterizing both the bacterial families present and their relative abundances in both cohorts before the onset of longitudinal observation. Following exclusion of unclassified reads and reads classified as chloroplasts, mitochondria, or non-prokaryotes, we found that a small number of families dominated the intestinal microbiome. Of 55 classifiable families, 14 families comprised 97.28% of the intestinal microbiome of October cohort bats (Fig. 1a). Similarly, the baseline microbial community composition of the May cohort bats was also dominated by a small number of bacterial families (Fig. 1a): only 11 families had relative abundances greater than 0.5% across the 30 May cohort bats, with 79 total families categorized as “other”. Dominant families across both cohorts included *Moraxellaceae*, *Enterobacteriaceae*, *Peptostreptococcaceae*, *Clostridiaceae*, *Leuconostocaceae*, *Mycoplasmataceae*, *Neisseriaceae*, *Streptococcaceae*, *Pseudomonadaceae*, and *Pasteurellaceae*. We also observed individual variation in microbial community composition, regardless of sex and cohort.

### Microbial community comparison of October and May cohorts

We then compared both the microbial community composition and diversity of the two cohorts, aggregating individuals to the cohort level, with the goal of determining whether they should be analyzed separately. In comparing alpha diversity metrics (Supplementary Table S1) between the two groups, the May cohort had slightly, but not significantly, higher median Shannon entropy and Simpson’s diversity scores than did the October cohort (Fig. 1b). The May cohort samples also had a median Faith’s phylogenetic diversity score of 4.98, as compared to the October cohort median of 3.98 (p = 0.0012, Mann–Whitney test). Taken together, these results indicate greater phylogenetic diversity, but not necessarily richness or evenness, in the intestinal microbiome composition of the May cohort bats as compared to the October cohort bats. Interestingly, this was despite the October cohort bats having a median number of observed features of 764, whereas the May cohort bats only had a median of 317.5 observed features (p = 0.0007, Mann–Whitney test). Thus, though the October cohort samples potentially had a larger total number of species than did the May cohort samples, the May cohort samples likely had greater phylogenetic diversity in those species that were present.

Several bacterial families that were abundant (i.e., percent composition greater than 0.5% of the microbiome) in the October cohort were not abundant in the May cohort. These families included *Sphingomonadaceae*, *Burkholderiaceae*, *Chitinophagaceae,* and *Spirochaetaceae*. We observed a new family, *Carnobacteriaceae*, in the May cohort samples. Among the families that remained abundant in both cohorts, there were differences in relative abundance between the two cohorts. *Moraxellaceae*, *Enterobacteriaceae*, and *Peptostreptococcaceae* were relatively more abundant in the October cohort, whereas *Clostridiaceae*, *Mycoplasmataceae*, and *Streptococcaceae* were more enriched in the May cohort.

We found significant (p = 0.001, PERMANOVA) community composition differences (Supplementary Table S1) in the intestinal microbiome between the two cohorts when using unweighted UniFrac (Fig. 1c, left) distance as well as Bray–Curtis (p = 0.001, PERMANOVA) and weighted UniFrac (p = 0.001, PERMANOVA) distance (Supplementary Fig. 2a,b). We conducted a differential abundance analysis and identified 21 bacterial families that displayed significantly (p < 0.05, LEfSe) different abundance between the cohorts (Fig. 1c, right). Concordant with our findings that the October cohort samples had higher median numbers of species, the October cohort was differentially enriched in 17 of the 21 families while the May cohort samples were relatively more abundant in only four. As expected, the *Carnobacteriaceae* family was differentially abundant in the May cohort. The other differentially abundant families also aligned with our initial observations of visually apparent abundance differences in microbial community composition in the two cohorts.

### Sex and pregnancy status differences in microbial community composition and diversity

**Figure 2:**
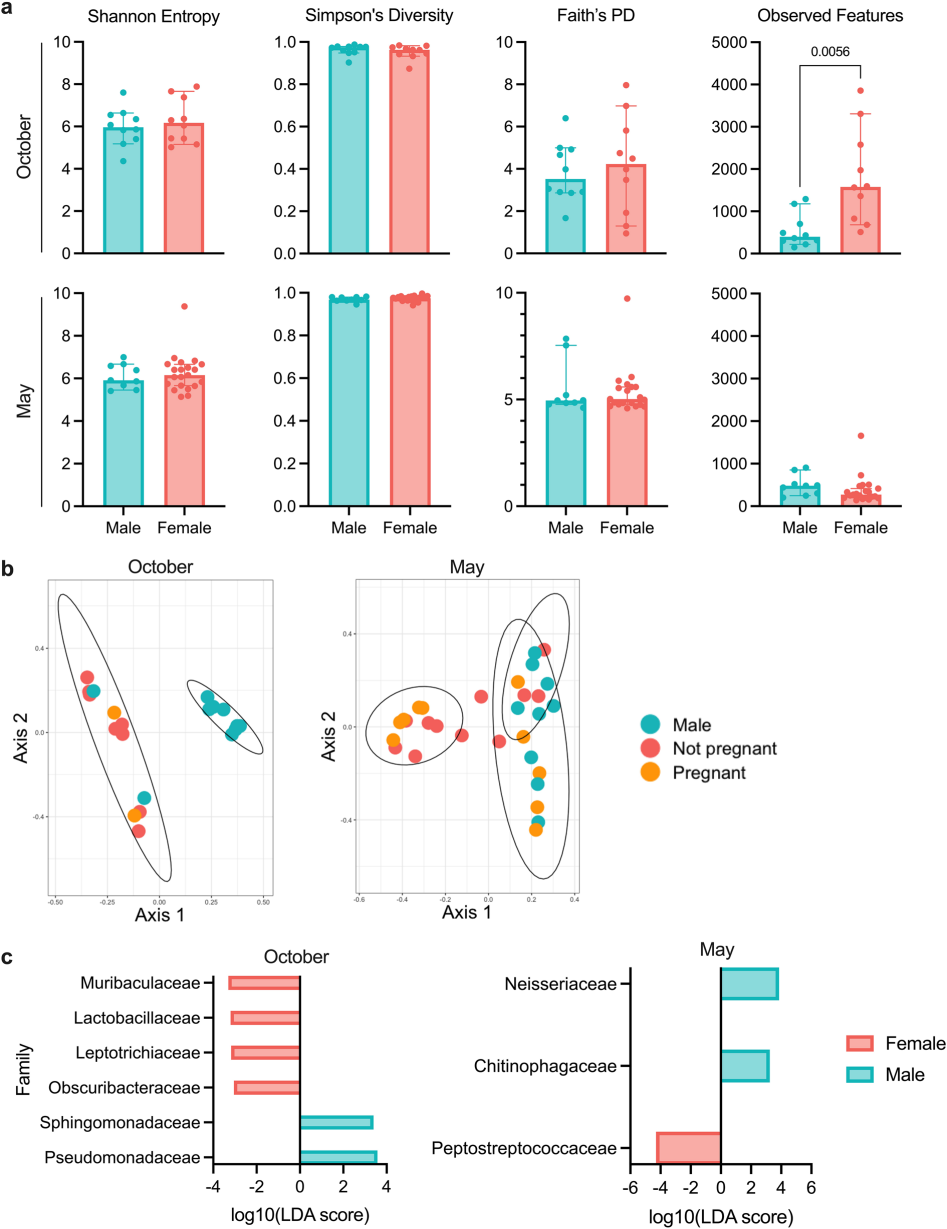
Intestinal microbial community composition and diversity of October and May cohort bats by sex (male, N = 10 October; N = 9 May and female, N = 10 October; N = 21 May) and pregnancy status (pregnant, N = 2 October; N = 10 May and not pregnant, N = 8 October; N = 11 May). (**a**) Alpha diversity metrics (left to right: Shannon entropy, Simpson’s diversity, Faith’s phylogenetic diversity, and observed features) of the October (top) and May (bottom) cohort intestinal microbiome by sex. Median and 95% confidence intervals with individual points overlaid. Significant p-values indicated; Mann–Whitney test (N = 10, October; N = 9 (males) and 21 (females), May). (**b**) Principal coordinate analysis showing unweighted UniFrac distance by sex and pregnancy status in the October cohort (left), and Bray–Curtis distance by sex and pregnancy status in the May cohort (right). Ellipses denote significant (kmeans, p < 0.05) clusters. Points colored by sex and pregnancy status. (**c**) log_10_(linear discriminant analysis score) of differentially abundant families (LEfSE, p < 0.05) between male and female bats at intake in the October (left) and May (right) cohorts.

Given the substantial and significant differences between the two cohorts in both microbial community composition and diversity, we chose to analyze the October and May cohorts independently rather than combining them. Considering documented mammalian sex and pregnancy status differences in immunity^42–44^, we analyzed the intestinal microbiota of each cohort by sex and pregnancy status. The intestinal microbiomes of female bats in the October cohort had marginally, but non-significantly, higher median Shannon entropy (female = 6.17; male = 5.97) and Faith’s phylogenetic diversity (female = 4.23; male = 3.52) values than did those of males (Fig. 2a). Female intestinal microbiomes had a median of 1578.5 observed features while male intestinal microbiomes had a median of 397.5 observed features (p = 0.0056, Welch’s t-test); however, female intestinal microbiomes displayed slightly—but not significantly—lower median Simpson’s diversity values than did males’ (female = 0.963; male = 0.975). Thus, female bats in the October cohort had larger average numbers of species in their intestinal microbiomes than male bats, but may have had lower evenness or phylogenetic relatedness, indicating potential dominance of a small number of families.

We observed significant sex (p = 0.001, PERMANOVA) and pregnancy status (p = 0.001, PERMANOVA) differences in the microbiome comparing male and female bats in the October cohort when we calculated unweighted UniFrac distance (Fig. 2b). Sex-related community composition differences were also observed via weighted UniFrac distance (p = 0.002, PERMANOVA), but not Bray–Curtis distance (Supplementary Fig. 3a,b). We identified six families that significantly contributed (p < 0.05, LEfSe) to the observed sex differences in community composition in the October cohort (Fig. 2c): *Muribaculaceae*, *Lactobacillaceae*, *Leptotrichiaceae*, *Obscuribacteraceae*, *Sphingomonadaceae*, and *Pseudomonadaceae*. Of these families, four (*Muribaculaceae*, *Lactobacillaceae*, *Leptotrichiaceae*, and *Obscuribacteraceae*) contributed to less than 0.5% each of the overall microbial community composition in the October cohort. All four of the rare families were differentially abundant in female bats, and both relatively abundant families were more abundant in male bats, further supporting our observation that October cohort female bats likely had a larger number of species overall but lower evenness amongst species than did male bats.

Unlike in the October cohort, we observed no sex differences in microbial diversity via any of Shannon entropy, Simpson’s diversity, Faith’s phylogenetic diversity, or observed features among the May cohort bats (Fig. 2a), though one female bat’s intestinal microbiome (Bat 30) displayed anomalously high diversity across all metrics. However, we did observe differences in microbial community composition, measured by Bray–Curtis distance, by both sex (p = 0.004, PERMANOVA) and pregnancy status (Fig. 2b; p = 0.032, PERMANOVA). This sex-associated community composition difference was present when the data were analyzed via unweighted (p = 0.047, PERMANOVA), but not weighted, UniFrac distance (Supplementary Fig. 4a). We identified three families that significantly contributed (p < 0.05, LEfSe) to observed sex differences after acclimation (Fig. 2c): *Neisseriaceae*, *Chitinophagaceae*, and *Peptostreptococcaceae*. Interestingly, there was no overlap between the two cohorts in those bacteria that contributed to sex differences in microbial community composition. Furthermore, the October cohort had more pronounced sex differences overall, as well as a larger total number of families that significantly contributed to sex differences.

### Longitudinal analysis of the bat intestinal microbiome

Next, we investigated changes in the intestinal microbiomes of May cohort bats over a 28-day period (Supplementary Table S2), finding that the beginning (day 0 (D0)) and end of the study period (day 28 (D28)) showed the most substantial differences compared to any of the intermediate timepoints (days 1, 4, 7, and 14 (D1, D4, D7, D14)). Thus, we began by comparing samples taken immediately after acclimation (D0; a subset of May cohort bats, N = 5 females and 4 males) and after 28 days (D28, N = 5 females and 4 males) to assess potential longitudinal changes in microbial diversity and community composition under the repeated isoflurane treatment required to obtain the samples. We also analyzed potential differences in longitudinal changes related to sex, pregnancy status, and housing scheme in our facility.

**Figure 3:**
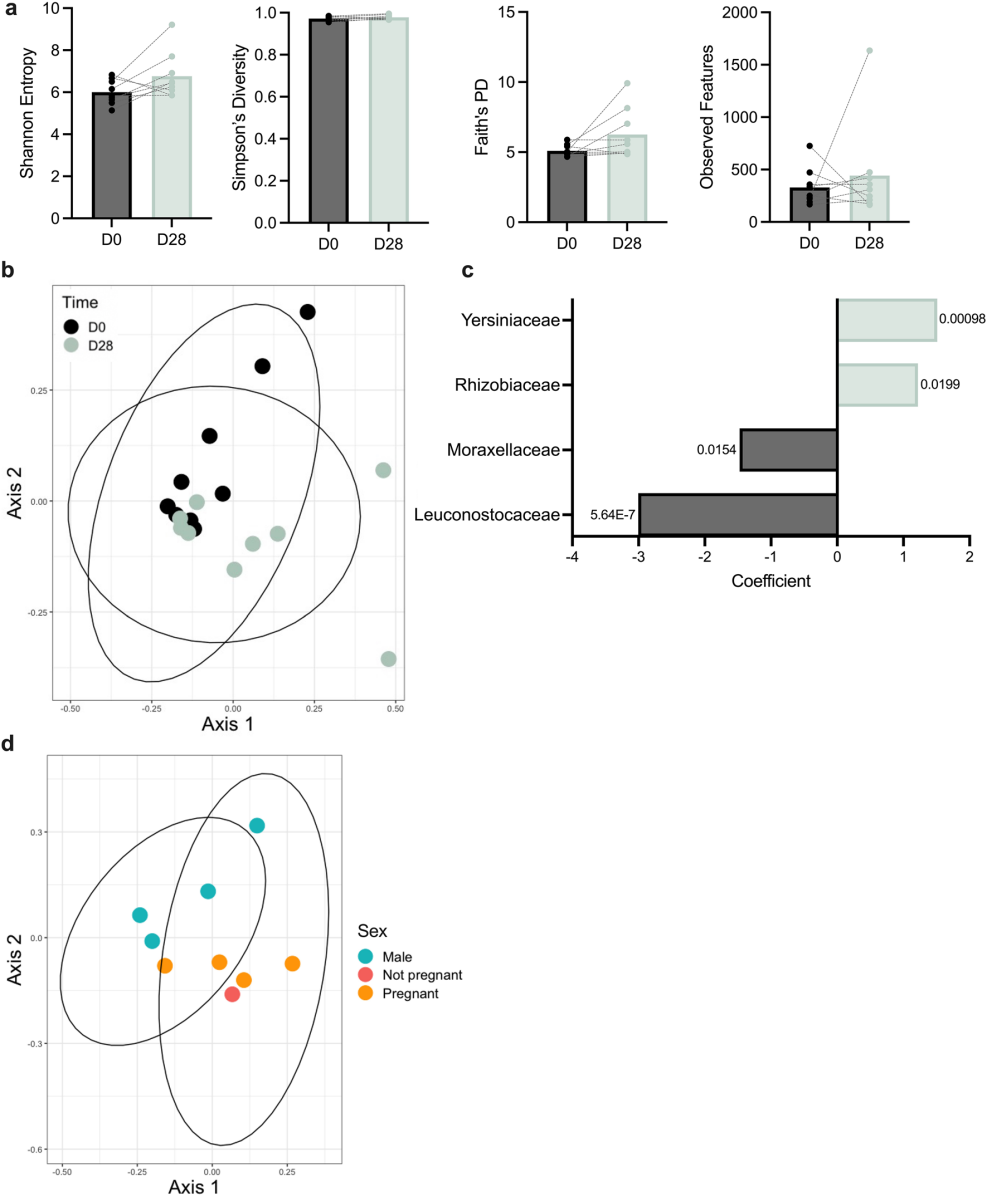
Intestinal microbial community composition and diversity of May cohort bats at study beginning (D0, N = 9) and end (D28, N = 9). (**a**) Alpha diversity metrics (left to right: Shannon entropy, Simpson’s diversity, Faith’s phylogenetic diversity, and observed features) of the intestinal microbiome. Median and 95% confidence intervals with individual points overlaid. Gray lines indicate points belonging to the same animal. Significant p-values indicated; Wilcoxon matched pairs test of bats euthanized at study endpoint (N = 9). (**b**) Principal coordinate analysis showing unweighted UniFrac distance between baseline and endpoint samples. Ellipses denote significant (kmeans, p < 0.05) clusters. Points colored by sampling time point. (**c**) Bar plot showing MaAsLin2 coefficients for families with corrected p-values < 0.05. Coefficients are equivalent to log_2_(fold change) over time; negative coefficients indicate decline in relative abundance and positive coefficients indicate increase in relative abundance. p-values corrected using Benjamini–Hochberg procedure shown. (**d**) Principal coordinate analysis showing Bray–Curtis distance by sex and pregnancy status at endpoint in the May cohort. Ellipses denote significant (kmeans, p < 0.05) clusters. Points colored by sex and pregnancy status.

Bats experienced slight, but non-significant by Wilcoxon matched pairs test, longitudinal increases in both the overall number of species present in their intestinal microbiomes as well as evenness and phylogenetic diversity of their microbial communities (Fig. 3a). Shannon entropy increased from a median of 5.83 at the beginning of the study to a median of 6.45 at the end of the study. Likewise, median Faith’s phylogenetic diversity increased from 4.99 to 5.58 and median observed features increased from 251 to 309. Finally, Simpson’s diversity increased slightly from a median of 0.972 at the beginning of the study to a median of 0.975 by the endpoint.

Microbial community composition, as measured by unweighted UniFrac distance, changed significantly (p = 0.016, PERMANOVA) (Fig. 3b). Neither the weighted UniFrac distance (Supplementary Fig. 5a) nor the Bray–Curtis distance (Supplementary Fig. 5b) captured this trend. To further investigate longitudinal changes in gut microbial composition, we tested the association between day and the relative abundance of families using MaAsLin2. Briefly, the log-transformed relative abundance of all families present in > 10% of the total number of samples were correlated with experimental day using a general linear model adjusting for sex and sample ID. Among 92 families tested, we identified four families that changed significantly by day (corrected p-values < 0.05, Fig. 3c), indicating longitudinal change over the duration of the study. Of these four families, *Moraxellaceae* and *Leuconostocaceae* decreased in relative abundance over time, whereas *Yersiniaceae* and *Rhizobiaceae* increased in relative abundance over time (Fig. 3c, Supplementary Fig. 7). Overall, the results suggest that time after acclimation influenced bat gut microbial composition to some extent.

We observed no microbiome convergence by cohousing scheme (Supplementary Fig. 6). Upon further inspection, we found that a small number of animals—though not the same animal each time—drove significant changes in relative abundance of different families (Supplementary Fig. 7). This appeared to be especially true for the *Sphingobacteriaceae* and *Bacillaceae* families, indicating the potential for a single bat to drive significant results in an entire group.

Observed sex differences were maintained through D28, with significant sex differences in microbial community composition observed via Bray–Curtis distance at D28 (Fig. 3d; p = 0.042, PERMANOVA). This was not observed with weighted and unweighted UniFrac distances (Supplementary Fig. 4b). No pregnancy status differences were observed at D28, likely due to both the small number of bats and the disproportionate number of pregnant females by the end of the study. Interestingly, though we observed statistically significant sex differences by Bray–Curtis distance, we were able to find no individual families that significantly contributed to these sex differences, indicating that these differences were likely due to several small changes in community composition.

### Intestinal metabolite analysis

Considering the persistent sex differences observed in microbial community composition and the microbiome’s links to intestinal metabolism, we investigated intestinal tract metabolite composition via ileal tissue sampling at the time of euthanasia (D28) for those bats we followed longitudinally (Supplementary Table S1). We were particularly interested in potential sex differences in metabolite composition, as well as the metabolome’s relation to the microbiome.

**Figure 4:**
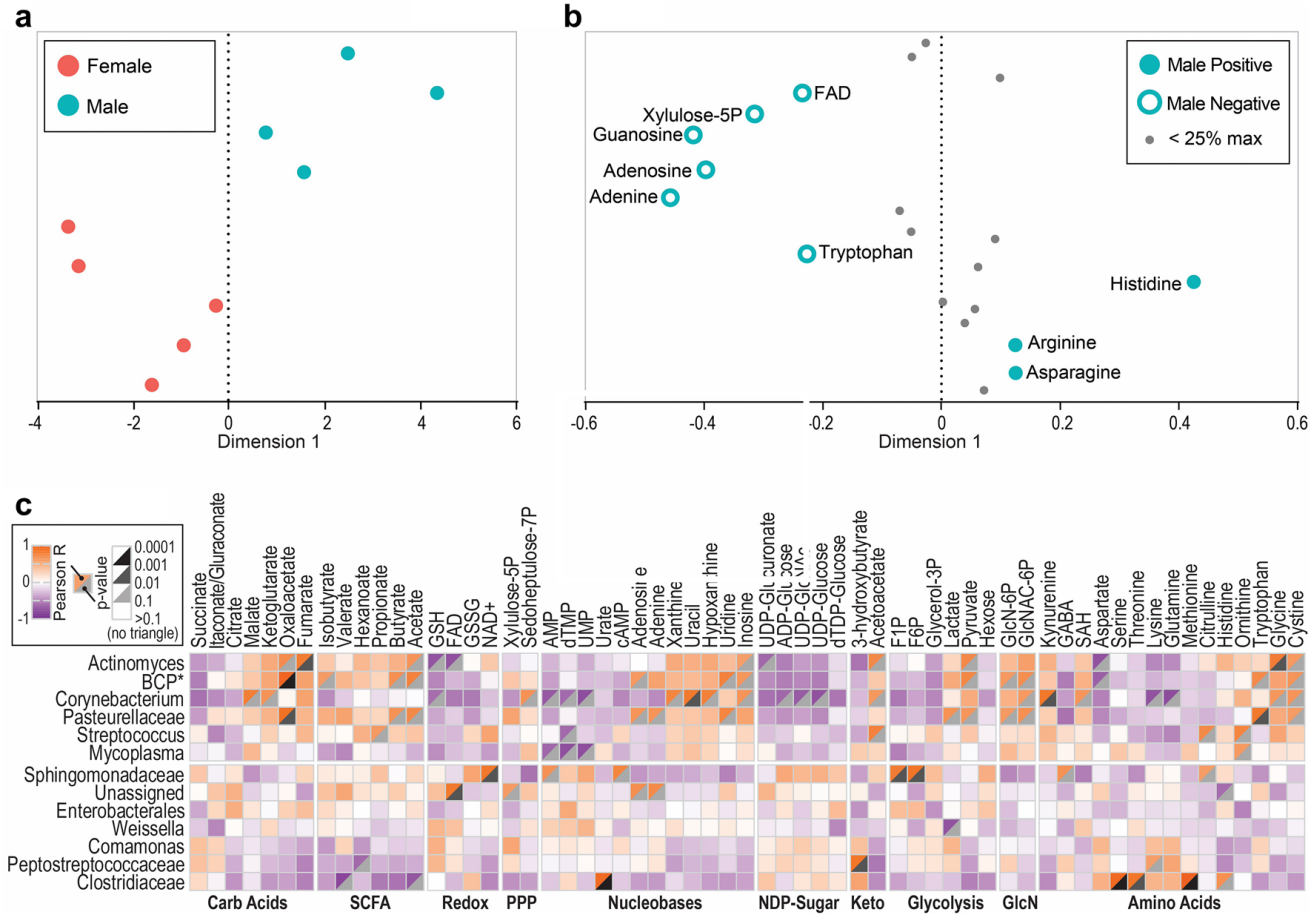
Metabolomics analysis of May cohort bats (N = 9). Metabolites were detected by mass spectrometry of intestinal samples collected during necropsy. (**a**) sPLSDA showing bat intestinal metabolomes by sex (female, N = 5 and male, N = 4) based on differences in relative metabolite abundance. Points colored by sex. (**b**) Loadings of sPLSDA analysis of metabolite differences according to sex. Metabolites greater than or equal to 25% of the maximum absolute loading value are colored according to the direction in which they contribute to intergroup differences and labeled with metabolite name. Metabolites less than 25% of the maximum absolute loading value are shown in gray and unlabeled. (**c**) Correlation plot showing R values and p-values (shown for significance 0.1 and under) for Pearson correlation between metabolites (columns) and bacterial genera (rows). Positive R values in orange; negative R values in purple. Large p-values in white; small p-values in black. Metabolites organized and labelled by family. Asterisk (*) denotes an abbreviation for the Burkholderia–Caballeronia–Paraburkholderia family.

In concordance with our microbiome data, we observed intestinal metabolite composition differences by sex (Fig. 4a), with male and female bats showing separation along dimension 1 of our sPLSDA analysis. Several metabolites contributed to this difference; asparagine (p = 0.1905, Mann–Whitney test) and arginine (p = 0.1111) were non-significantly enriched and histidine (p = 0.0159) was significantly enriched in males, while the nucleobases adenine (p = 0.0317), adenosine (p = 0.0317), and guanosine (p = 0.0317) were all significantly enriched in females (Fig. 4b, Supplementary Fig. 8).

To identify primary bacterial drivers of the metabolic state, we calculated Pearson correlation coefficients of intestinal metabolite profiles with microbiome genus abundance (Fig. 4c). A cluster of bacterial genera, including *Actinomyces, Burkholderia–Caballeronia–Paraburkholderia, Corynebacterium, Pasteurellaceae, Streptococcus*, and *Mycoplasma* correlated with a shared metabolic pattern that was consistent across multiple metabolite families. In general, this group of bacteria was positively correlated with the levels of SCFAs, certain nucleobases and carboxylic acids, pyruvate and lactate, and specific amino acids, such as tryptophan, cystine, and glycine. These bacteria were negatively associated with the levels of flavin and nicotinamide coenzymes, NDP-sugars, and the amino acids aspartate, glutamine, and lysine.

The dominant *Actinomyces*-associated metabolic pattern was anticorrelated with a cluster of genera including *Clostridiaceae, Peptostreptococcaceae, Comamonas, Weissella, Enterobacterales*, and *Sphingomonadaceae*. In contrast to the other cluster of bacteria, these bacteria were correlated with higher levels of glycolytic intermediates, aspartate, and ketone bodies. *Clostridiaceae* was especially closely associated with increased levels of serine and methionine in the intestine. Finally, independent of any major metabolic patterns observed, *Sphingomonadaceae* was found to be associated with metabolites related to carbohydrate metabolism, and specifically low levels of metabolites produced as a result of glucosamine metabolism.

## Discussion

We investigated community composition and diversity of the intestinal microbiome of two cohorts of captive Jamaican fruit bats, one of which we followed longitudinally over a 28-day period. Through Illumina sequencing and analysis of rectal swabs, we established a novel method for microbiome analysis of samples removed from high and maximum containment. Though many viruses of interest require BSL-4 conditions, such analyses were not previously possible due to limited availability of inactivation methods approved for these conditions^45–47^. The incorporation of acceptable inactivation methods for samples obtained in containment into our methodology will allow for analysis of bat samples during infection with BSL-4 viruses. Furthermore, our use of rectal swabs for longitudinal sampling of each bat facilitates time-course studies during infection and enables the comparison of each bat to its own initial baseline. Together, the ability to longitudinally analyze changes in both the microbiome and metabolome over the course of infection enables detailed studies of their interaction and the potential effects on immunological responses to infection in bats.

Surprisingly given consistency in housing and diet, we observed individual variation in microbial community composition across both cohorts. In humans, the microbiome displays individual variation associated with health-related host phenotypes^48^. In primates, individual variation in the intestinal microbiome is related to social structures and groups rather than habitat or diet, with adult microbiomes varying more with group affiliation than environment^49^. Furthermore, work on wild-caught vampire bats (*Desmodus rotundus*) that were merged into one colony upon capture suggests that social interactions influence the intestinal microbiome even when controlling for other factors^50^. Wild Jamaican fruit bats form harem groups composed of multiple females and a single male^51,52^, which may influence their intestinal microbiomes in ways that are difficult to observe.

Importantly, most of these animals harbored the same few dominant bacteria despite variation in their abundances. This aligns with the observation that most mammalian species possess a “core” microbiome, defined as the set of microbial taxa characteristic of that species^53^. Laboratory mice harbor the same core bacterial families across individuals and even species^54^, though they may experience less variation due to inbreeding. Future work corroborating these observations in other populations of Jamaican fruit bats is necessary to assess the presence or absence of a species-wide core microbiome.

We observed differences in microbial community composition and diversity between our two cohorts after acclimation, despite previous work suggesting convergence of the microbiome of various wild bat species as a result of captivity^55,56^. In particular, the May cohort appeared to have a smaller number of total bacterial species but increased phylogenetic diversity between species than did the October cohort. The two cohorts also had phylogenetically distinct microbiomes. Given evidence indicating that functional traits may be conserved in phylogenetically similar bacteria^57^, the microbiome may be serving different functions in the two cohorts, with the May cohort microbiome potentially having broader functionality. Though reproductive status may drive seasonality of the intestinal microbiome^58–60^, the captive colony from which these cohorts were formed anecdotally experiences no observable reproductive seasonality, ruling out birth pulses as a driver of cohort-level community composition differences.

However, we propose several potential explanations for cohort-level differences in microbial community composition. Despite these cohorts lacking exposure to seasonal changes in their environment, long-standing adaptations to seasonality in fruiting and flowering patterns may have driven seasonality in the microbiome that is retained in captivity, explaining differences between the two cohorts. Diet affects the intestinal microbiome^61–63^; though diets were kept constant between the two cohorts, seasonal differences in sourcing and availability of fruit may also have contributed to our observations. Though there are limited data on the effects of shipment on mammalian intestinal microbiomes, the microbiomes of these two cohorts may have been affected differently by the process of shipment and facility transfer. Though transport conditions were controlled and under strict animal welfare regulations, environmental conditions (temperature, light cycling, etc.) during transit may have varied slightly by season, potentially contributing to differences in the microbiomes of the cohorts upon arrival. Alternatively, data show humanization of the microbiome in captive animals, potentially indicating transfer of intestinal bacteria between animals and their human caretakers^64–66^. Thus, seasonal variation in caretaker microbiomes may have influenced differences between the intestinal microbiomes of our two cohorts. However, all caretakers and investigators were always wearing full personal protective equipment, reducing the likelihood of microbiome transfer. More likely, seasonal variation in the sourcing and availability of fruit, these bats’ primary food source, contributed to differences between the October and May cohorts. Future studies should consider methods such as irradiation of provided food or specific decontamination procedures for care staff to mitigate these risks. Regardless, our observations of individual and cohort variation in community composition highlight the importance of establishing baseline values for both individuals and cohorts in future studies.

Despite these cohort-level differences, several dominant bacterial families in both cohorts, including *Enterobacteriaceae*, *Moraxellaceae*, and *Streptococcaceae*, comprise the microbiota of wild rousette and pteropid bats^67–70^, potentially signaling a pattern of dominance of a few families across multiple bat species. Several of these families, including *Enterobacteriaceae* and *Moraxellaceae*, belong to the Pseudomonadota phylum, the abundance of which may indicate microbial dysbiosis in humans^31,71^. However, this microbial signature is thought to contribute to decreased inflammatory responses observed in human centenarians^72–75^, providing a potential contributor to bats’ posited immune tolerance.

We demonstrated significant longitudinal changes to microbial diversity and community composition over a 28-day period with multiple anesthetic events. As in the cohort-level differences, the difference in unweighted UniFrac distance at D0 and D28 implies phylogenetic, and thus potentially functional, changes to the intestinal microbiome over this period. This exposure to a new environment likely introduced new microbes to the bats’ intestinal tracts, increasing diversity; similar increases in diversity have been observed in wild bats introduced to a new environment in captivity^55^. These changes demonstrate the importance of including a longitudinal microbiome control in future studies.

We observed that a small group of bacteria, including *Actinomyces* and *Streptococcus*, was correlated with increased levels of SCFAs. While not typically considered primary producers of SCFAs, *Actinomyces* and *Streptococcus* are positively associated with systemic SCFA levels in human obesity models^76^, and *Streptococcus* can produce acetate, a SCFA, via the Wood-Ljungdahl pathway^77^. The presence of this association in bats may indicate either that these species are producers of SCFAs, interaction with other species encourage them to produce SCFAs, or that the colonization of these species is encouraged by SCFAs. Various SCFAs have been repeatedly demonstrated to potentially be involved in immunoregulation and gut-brain communication in mouse models^78–80^, and butyrate has been implicated as enhancing antibody responses of murine splenocytes^81^, indicating the possibility of a similar function in bats.

We also found associations between bacteria and tryptophan levels in our data. *Streptococcus,* which was associated with increased tryptophan, can produce serotonin from tryptophan^82^. *Clostridiaceae*, which was associated with decreased tryptophan, can decarboxylate tryptophan^83^. These data indicate a potential role of these bacteria in regulating tryptophan levels. In humans, tryptophan is associated with intestinal immunity, with alterations being linked to disease and the intestinal microbiome^84^. Thus, concordant with previous literature describing humans and mice^85–87^, the intestinal microbiome of Jamaican fruit bats may drive metabolome changes that affect intestinal immunity and immunoregulation.

We observed sex differences in microbial community composition measured by unweighted UniFrac, but not Bray–Curtis distance, in both cohorts, indicating that male and female bat microbiomes may have substantial overlap in species present, but that non-overlapping species are phylogenetically distant. This result highlights the need for more work investigating the roles of microbes not shared by male and female bats in order to further elucidate sex differences.

Regardless, these sex differences align with observed differences in microbial diversity and community composition based on reproductive stage and pregnancy status in lesser long-nosed bats (*Leptonycteris yerbabuenae*)^60^. Prior work suggests that when sex differences in the intestinal microbiome are present, female bats have more diverse microbiomes than males^59,60,88^, though our observations on this relationship were equivocal. We also observed the maintenance of the initially observed sex differences over the study period. However, the factors contributing to these sex differences changed between the study’s onset and its end, indicating that male and female microbiomes may react differently to stimuli. This finding is consistent with observations in mice indicating that aging leads to sex-specific remodeling of the intestinal microbiome^89^ and that diet- and stress-induced cecal and fecal microbiome changes are sex-dependent^90^.

As with our observations in the microbiome, we found sex differences in the intestinal metabolome. The metabolomes of various bodily fluids and tissues have been shown to respond to stimuli and disease states in a sex-dependent manner across a range of species^91–93^, suggesting that sex differences in the metabolome are persistent and may be linked to health status. Thus, further work on sex differences in the intestinal microbiomes and metabolomes of Jamaican fruit bats will be useful for better understanding sex differences in various health states, including immune responses to viral infections. To our knowledge, this study is one of the first to link microbial community composition to function via metabolites, thus providing a foundation for further research building on the functional role of the bat intestinal microbiome.

Limitations of this study included the fact that we only followed nine bats longitudinally, and of five longitudinally studied females, four were pregnant. These bats were not age-matched; as age may affect microbiome composition, especially during infection studies^34,94,95^, this could contribute to the noise in our data, as could the fact that the pregnancies observed were not synchronized. Finally, captive bats may not be representative of a population of wild bats. Though experiments in the lab will never fully recapitulate natural phenomena, these experimental studies provide a controlled setup to longitudinally assess immune responses.

The human microbiome has been explored as a predictor for immune-related disease outcomes, including response to rheumatoid arthritis treatment and mortality in allogeneic hematopoietic-cell transplantation^96,97^. Specific metabolites already reliably predict certain conditions or disease states in humans, including prediabetes and diabetes mellitus^98^. Few studies in bats have combined microbiome analysis with metabolomics; we hope this study provides a foundation for further investigation of the functional role of the bat intestinal microbiome, allowing for prediction of the results of changes in the microbiome. Given our initial work on associations between bacteria, metabolites linked to intestinal immunity and immunoregulation, and health states (i.e., reproductive status) in this study, intestinal microbial composition could eventually be used to infer disease states and outcomes.

We recognize the logistical challenges inherent in longitudinally sampling and conducting infection studies in bats, especially wild bats. However, accounting for individual variation, potential seasonality, and life course differences in the microbiome will allow for more robust microbiome studies in both captive and wild bats. The methodology described here establishes a foundation for future infection studies conducting immunological assessments of the intestinal microbiomes of Jamaican fruit and other bats, including with viruses that must be handled under high containment conditions. Finally, the integration of metabolomics with microbiome data will allow for more detailed studies of the functional role of the microbiome and the effects of microbiome changes on immunity with the intestinal metabolome as a mediator, potentially leading to the bat intestinal microbiome as an effective predictive tool for health and disease states.

## Materials and methods

### Ethics statement

All animal experiments were conducted in an AAALAC International-accredited facility and were approved by the Rocky Mountain Laboratories (RML) Animal Institutional Care and Use Committee following the guidelines put forth in the Guide for the Care and Use of Laboratory Animals 8th edition, the Animal Welfare Act, United States Department of Agriculture and the United States Public Health Service Policy on the Humane Care and Use of Laboratory Animals. The study is reported in accordance with ARRIVE guidelines.

### Biosafety

All experiments were conducted as if under BSL-3 or BSL-4 conditions as approved by the Institutional Biosafety Committee (IBC). For the removal of specimens from high containment areas, inactivation of all samples was performed according to IBC-approved standard operating procedures.

### Animals

Mixed-sex healthy young adult to adult Jamaican fruit bats were transferred from Colorado State University (CSU)’s closed colony to RML (approx. weight range = 32.3 g to 55.5 g upon intake at RML). At CSU, a colony of several hundred bats was housed in an open mixed-sex free-flight facility^99^. No previous procedures were performed on any animal, and animals were not genetically modified or immunosuppressed. The temperature range was kept between 22 and 26 °C, with humidity between 30 and 75%. The enclosure’s floors, walls, and equipment were sanitized biweekly. Due to (1) no prior availability of microbiome data on which to base a power analysis for this species and (2) the observational and descriptive nature of this study, no power calculation was performed to determine group sizes. For similar reasons, alongside the lack of an explicit treatment group, each animal’s own baseline is used as its reference point, and no negative control group was included. Animals were randomly selected for enrollment from the full colony at CSU. After transport, animals were randomly housed in new cage groups and kept separated by sex. For all longitudinal sampling, the subset of sampled animals was randomly assigned prior to study start. No animals were excluded after enrollment. Pentobarbital sodium and phenytoin sodium after isoflurane inhalation was used for euthanasia of the October cohort; bilateral thoracotomy after isoflurane inhalation was used for euthanasia of the May cohort.

A first shipment (October 2021) consisted of 20 bats, 10 male and 10 female, of which two were pregnant. The second shipment (May 2022) consisted of 30 bats, 9 male and 21 female, of which 10 were pregnant (Supplementary Table 1). Physical exams were performed on all bats at intake, and bats were cohoused in our facility in sex-separated groups of up to five bats per cage. Cages were cleaned with water and cage pans disinfected daily with 5% Micro-Chem Plus Detergent Disinfectant (NCL). Bats were provided a diet of various fresh, non-citrus fruits supplemented with 15 g of Mazuri Softbill Diet (Mazuri) per 10 bats daily. Fruit was provided ad libitum and replaced twice daily. Temperature and humidity were kept constant and in the same range as the CSU facility. Bats were allowed a minimum of 5 days to acclimate to the facility prior to study onset. All cages were housed within the same room and provided the same diet to avoid environmental confounding. Any environmental differences that may have occurred between October and May, as well as seasonal availability and sourcing of fresh fruit, were not controlled for beyond what was described above. Animal care staff and experimenters were aware that bats belonged either to the October or May cohort. Sequencing and downstream analysis were performed blinded until results were allocated to the metadata.

### Cross-sectional microbiome sample collection

Rectal swabs and fecal samples were obtained from 20 bats in the October cohort, and rectal swabs were obtained from 30 bats in the May cohort after a 5- or 6-day acclimation period but within a week of their arriving at our facility. Swabs and fecal samples were either frozen immediately after collection or transferred to TRIzol (ThermoFisher) and stored at − 80 °C. All swabs were collected using pre-wetted Puritan 6″ Sterile Mini-Tip Polyester Swabs with Ultrafine Polyester Handles (Puritan) under the influence of 5% isoflurane anesthesia.

A subset of samples was used to establish the methods for microbiome sampling and analysis of samples coming from maximum containment (BSL-4) conditions. Due to limited availability of bat samples, these methods were first tested and optimized using feces collected from cages of an in-house breeding colony of multimammate rats (*Mastomys natalensis*) maintained at our facility^100^. Fecal samples were stored at – 80 °C until use. Nucleic acid extraction was performed using one of TRIzol Reagent (ThermoFisher), the AllPrep DNA/RNA Kit (Qiagen), or the QIAamp Fast DNA Stool Mini Kit (Qiagen) per manufacturer’s instructions. The concentration of DNA was quantified with the NanoDrop 8000 (Thermo Scientific) before use in downstream applications.

### Metabolome and longitudinal microbiome sample collection

Swabs were collected from a subset of animals (N = 9; female, N = 5 and male, N = 4; pregnant, N = 4 and not pregnant, N = 1) in the May cohort for longitudinal microbiome analysis. Rectal swabs were taken from these bats on each of days 1, 4, 7, 14, and 28. On day 28, all nine animals were euthanized, necropsied, and a 1 cm sample of ileal tissue was collected from each bat for metabolome analysis.

### Nucleic acid extraction for cross-sectional and longitudinal microbiome analysis of rectal swab samples

Swabs were immediately transferred to TRIzol (Invitrogen) and frozen at − 80 °C until use. DNA was isolated using TRIzol reagent per manufacturer’s instructions with a final resuspension volume of 0.5 mL.

### Nucleic acid amplification

The V3 and V4 regions of the 16S rRNA gene were amplified via amplicon PCR using 12.5 μL 2× KAPA HiFi HotStart ReadyMix (Roche) and 5 μL each of established primers^101^ at 1 μM each with 2.5 μL of extracted DNA. Cycling conditions were as follows: 95 °C for 3 min, followed by 35 cycles of 95 °C for 30 s, 55 °C for 30 s, and 72 °C for 30 s. PCR cycling conditions were based on manufacturer’s instructions (Illumina) and modified for bat samples. Samples were then held at 72 °C for 5 min followed by 4 °C indefinitely. PCR products were run on 1% agarose gels at 120 V for 25 min to verify successful amplification of the desired region before sequencing and library preparation.

### Illumina sequencing of 16S rRNA gene

Each sample was indexed and cleaned for library preparation per manufacturer’s instructions (Illumina). Twenty-five microliters of supernatant from each sample was collected for sequencing. Samples were fragment-sized using either a BioAnalyzer DNA 1000 chip (Agilent, October cohort) or a TapeStation 4200 (Agilent, May cohort) and quantitated using KAPA Library Quant Kit (Illumina) Universal qPCR Mix (KAPA Biosystems). Samples were diluted and multiplexed into a single pool using equal volumes. For the May cohort samples, an initial nano flow cell on a MiSeq Sequencing System (Illumina), which sequenced 150 cycles in each read direction for a total of 300 cycles each, was run to normalize the sample pool. Finally, a titration with 7% PhiX was used to cluster one V3 flow cell on a MiSeq Sequencing System (Illumina), which was sequenced for 300 cycles in each read direction for a total of 600 cycles each.

### Microbiome data analysis

The Quantitative Insights into Microbial Ecology 2 (QIIME2) tool (version 2021.7; open-source software)^102^ pipeline was used to trim and quality filter sequences. Amplicon sequence variants (ASVs) were determined using Deblur^103^ and clustered into de novo operational taxonomic units (OTUs) at the 98% similarity level using VSEARCH^104^. Rarefaction curves to assess species richness and comparability of the samples were also generated through these pipelines. Chimeras were removed, and taxonomy was assigned to OTUs using a classifier built with Scikit-learn^105^ and verified against the Greengenes^106^ 16S rRNA gene database. A phylogenetic tree for diversity analyses was generated with the FastTree pipeline^107^. QIIME2 was used to process the dataset. As QIIME2 uses rarefaction as a built-in normalization method (to remove bias due to variable sequencing depths), downstream analyses—including ordination and differential abundance analysis—were performed on normalized data.

### Metabolite and lipid sample preparation

Liquid chromatography-mass spectrometry (LCMS) grade solvents were used for all LCMS methods. Tissue samples were immersed directly in 0.4 mL of methanol and shredded at 30Hz for 10 min using a tissue shredder and one stainless steel bead (Qiagen, 5 mm) per sample. Supernatant was then irradiated at 2 mRad for sample removal from high containment. Then, 0.4 mL of water and 0.4 mL of chloroform were added to each sample. Samples were shaken for 30 min at 4 °C and centrifuged at 16,000×*g* for 20 min to establish layering. Four hundred microliters of the top (aqueous) layer was collected. The aqueous layer was diluted 5× in 50% methanol in water for LCMS injection. A subaliquot of the aqueous layer was taken for *O*-benzylhydroxylamine derivatization of carboxylic acids and SCFA analysis.

### Short chain fatty acid derivatization

Samples were derivatized with O-benzylhydroxylamine (O-BHA) according to previously established protocols with modifications^108,109^. A reaction buffer consisting of 1 M pyridine and 0.5 M hydrochloric acid in water was prepared fresh. A volume of 35 µL of the aqueous metabolite extract was sub-aliquoted. Ten microliters of 1 M O-BHA in reaction buffer and 10 µL of 1 M 1-ethyl-3-(3-dimethylaminopropyl)carbodiimide in reaction buffer were added to the sample. Samples were shaken at room temperature for 2 h. The reaction was quenched with 50 µL of 0.1% formic acid for 10 min. Derivatized carboxylic acid compounds were extracted via the addition of 400 µL ethyl acetate. Following mixing and centrifugation at 16,000×*g* for 5 min at 4 °C to induce layering, the upper (organic) layer was collected and dried under vacuum. Samples were resuspended in 300 µL of water for LCMS injection.

### Liquid chromatography–mass spectrometry

Tributylamine and all synthetic molecular references were purchased from Millipore Sigma. Methanol, LCMS grade water, isopropanol, and acetic acid were purchased through Fisher Scientific. All samples were separated using a Sciex ExionLC™ AC system and measured using a Sciex 5500 QTRAP® mass spectrometer. Aqueous metabolites were analyzed using a previously established ion pairing method with modification^110^. Quality control samples were injected after every 10 injections. Samples were separated on a Waters™ Atlantis T3 column (100 Å, 3 µm, 3 mm × 100 mm) and eluted using a binary gradient from 5 mM tributylamine, 5 mM acetic acid in 2% isopropanol, 5% methanol, 93% water (v/v) to 100% isopropanol over 15 min. Two distinct MRM pairs in negative mode were used for each metabolite. Derivatized short chain fatty acid samples were separated with a Waters™ Atlantis dC18 column (100 Å, 3 µm, 3 mm × 100 mm) and eluted using a 6-min gradient from 5 to 80% B with buffer A as 0.1% formic acid in water and B as 0.1% formic acid in methanol. Short chain fatty acids and central metabolic carboxylic acids were detected using MRMs from previously established methods, and identity was confirmed by comparison to derivatized standards^108,109^. All signals were integrated using MultiQuant® Software 3.0.3. Signals with greater than 50% missing values were discarded, and remaining missing values were replaced with the lowest registered signal value. All signals with a QC coefficient of variance greater than 30% were discarded. Metabolites with multiple MRMs were quantified with the higher intensity MRM. Filtered datasets were total sum normalized prior to analysis. Short chain fatty acid datasets were stitched to their corresponding polar metabolite dataset via common signals for lactate.

### Data analysis

Percent abundance of microbial families in each sample was calculated and visualized in GraphPad Prism (version 9.3.1). Alpha diversity (observed features, Shannon entropy, Simpson’s diversity, and Faith’s phylogenetic diversity) of each sample was calculated in QIIME2. Alpha diversity was visualized, and statistical significance (Mann–Whitney tests for comparison of males to females and October to May cohorts, and Wilcoxon matched pairs tests for longitudinal analysis) calculated, using GraphPad Prism. Beta diversity (Bray–Curtis distance, and weighted and unweighted UniFrac distance) was calculated and visualized in R (version 4.1.3)^111^ through the RStudio interface (version 2022.02.1)^112^ using the ecodist^113^, factoextra^114^, mia^115^, and ggplot2^116^ packages. PERMANOVAs to assess statistical significance of microbial community composition differences were also performed in R using the vegan package^117^. LEfSe analysis^118^ was performed in Galaxy and visualized in GraphPad Prism to assess differential abundance across groups. Microbiome Multivariable Associations with Linear Models (MaAsLin2, version 1.12.0) was used to test associations between experimental days and microbial families in R 4.2.3^119^. Briefly, the unrarefied OTU table was collapsed to the family level, normalized to relative abundance, and log transformed. Families present in > 10% of the total number of samples were selected for the association test. General linear model (LM) was applied with including sex and sample ID as fixed effect and random effects, respectively. p-values were corrected using the Benjamini–Hochberg procedure. Single and multi-variate analysis on metabolomics data was performed in MarkerView® Software 1.3.1 or using the mixOmics R package^120^ and loadings and variates visualized in GraphPad Prism. Abundances of specific metabolites were visualized and analyzed in GraphPad Prism. Correlations between metabolites and bacterial genera were calculated and visualized in GraphPad Prism.

### Supplementary Information

Supplementary Information.

## Data Availability

Data to be deposited in Figshare under 10.6084/m9.figshare.24917568. The sequencing data are accessible on NCBI’s Sequence Read Archive under Accession Number SRP440894.
