# Supplementary material for: Sex differences and individual variability in the captive Jamaican fruit bat (Artibeus jamaicensis) intestinal microbiome and metabolome
Source: Sci Rep. 2024 Feb 9;14:3381. doi: 10.1038/s41598-024-53645-5 (PMC10858165; doi:10.1038/s41598-024-53645-5)
Supplement: Supplementary file 1 — Supplementary Information. [file 41598_2024_53645_MOESM1_ESM.pdf]

Supplementary information - Sex differences and individual variability in the captive Jamaican fruit bat (*Artibeus jamaicensis*) intestinal microbiome and metabolome

Authors: Jade C. Riopelle[1], Amirhossein Shamsaddini[2], Myndi G. Holbrook[2], Eric Bohrnsen[2], Yue Zhang[3], Jamie Lovaglio[4], Kathleen Cordova[4], Patrick Hanley[4], Lon V. Kendall[5], Catharine M. Bosio[6], Tony Schountz[5], Benjamin Schwarz[2], Vincent J. Munster[1], Julia R. Port[1]*

1. *Laboratory of Virology, Division of Intramural Research, National Institute of Allergy and Infectious Diseases, National Institutes of Health, Hamilton, MT, USA*
2. *Research Technologies Branch, Division of Intramural Research, National Institute of Allergy and Infectious Diseases, National Institutes of Health, Hamilton, MT, USA*
3. *Integrated Data Sciences Section, Research Technologies Branch, National Institute of Allergy and Infectious Diseases, National Institutes of Health, Bethesda, MD, USA*
4. *Rocky Mountain Veterinary Branch, Division of Intramural Research, National Institute of Allergy and Infectious Diseases, National Institutes of Health, Hamilton, MT, USA*
5. *Department of Microbiology, Immunology, and Pathology, Colorado State University, Fort Collins, CO, USA*
6. *Laboratory of Bacteriology, Division of Intramural Research, National Institute of Allergy and Infectious Diseases, National Institutes of Health, Hamilton, MT, USA*

* *Correspondence: JRP: julia.port@nih.gov, +1 406 880 9634*

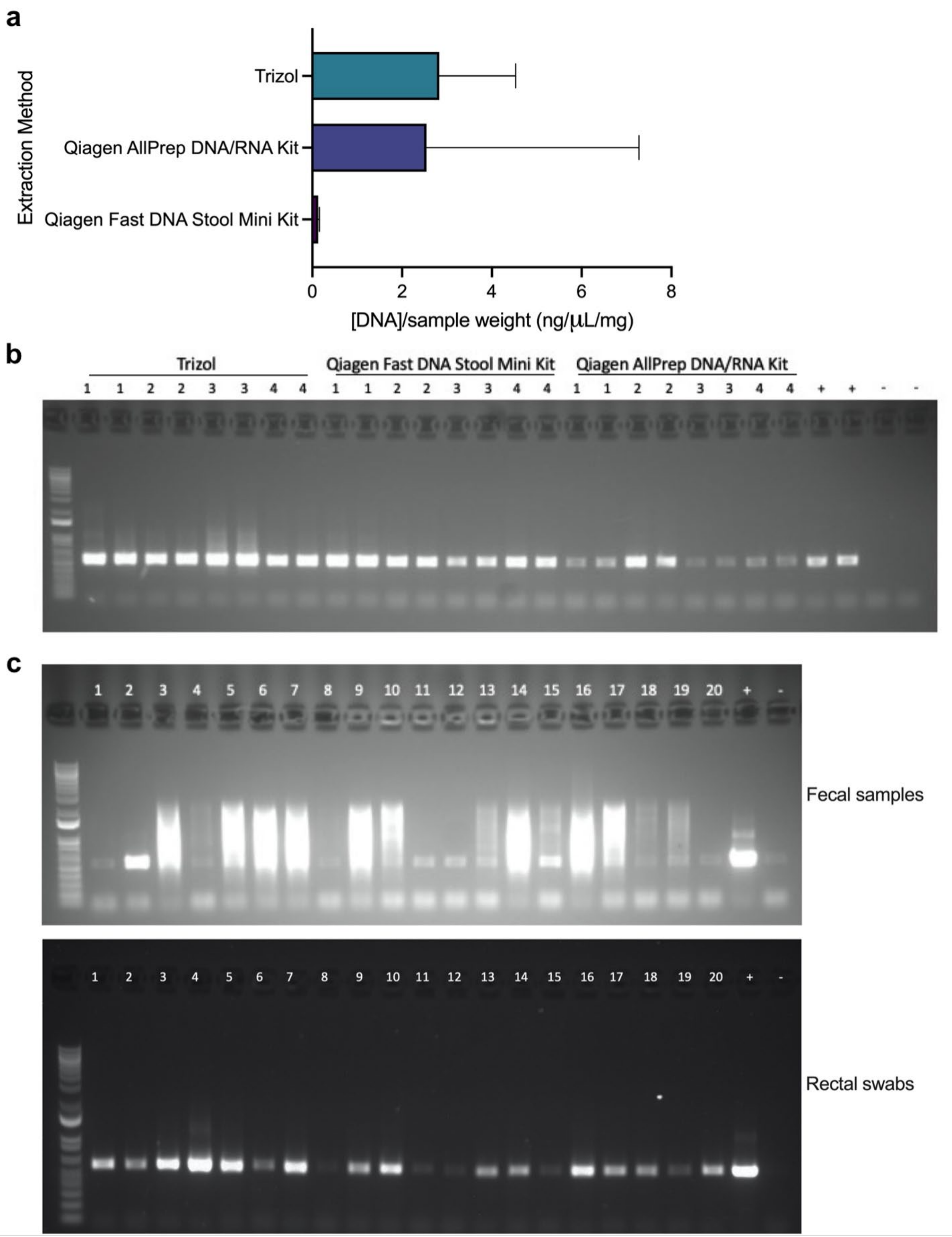

Supplemental Figure 1. Pipeline establishment for microbiome analysis of captive Jamaican fruit bats (*Artibeus jamaicensis*). **a.** Comparison of DNA yield of various nucleic acid extraction

methods. Due to limited sample availability, multimammate rat (*Mastomys natalensis*) fecal samples were used as a proxy for Jamaican fruit bat feces. Median with 95% confidence interval (N = 4). **b.** Gel electrophoresis showing DNA amplification results of multimammate rat feces extracted with different nucleic acid extraction methods, with 1Kb Plus DNA Ladder on the left (Invitrogen). Extracted *E. coli* DNA was used as positive control; water was used as negative control. **c.** Gel electrophoresis showing DNA amplification results of 10μL of Jamaican fruit bat fecal sample (top) and rectal swab (bottom) PCR products taken from a cohort of captive adult Jamaican fruit bats. 1Kb Plus DNA Ladder is on the left. Complete gels are depicted with the edges cropped. Fecal and rectal samples were run on two separate gels.

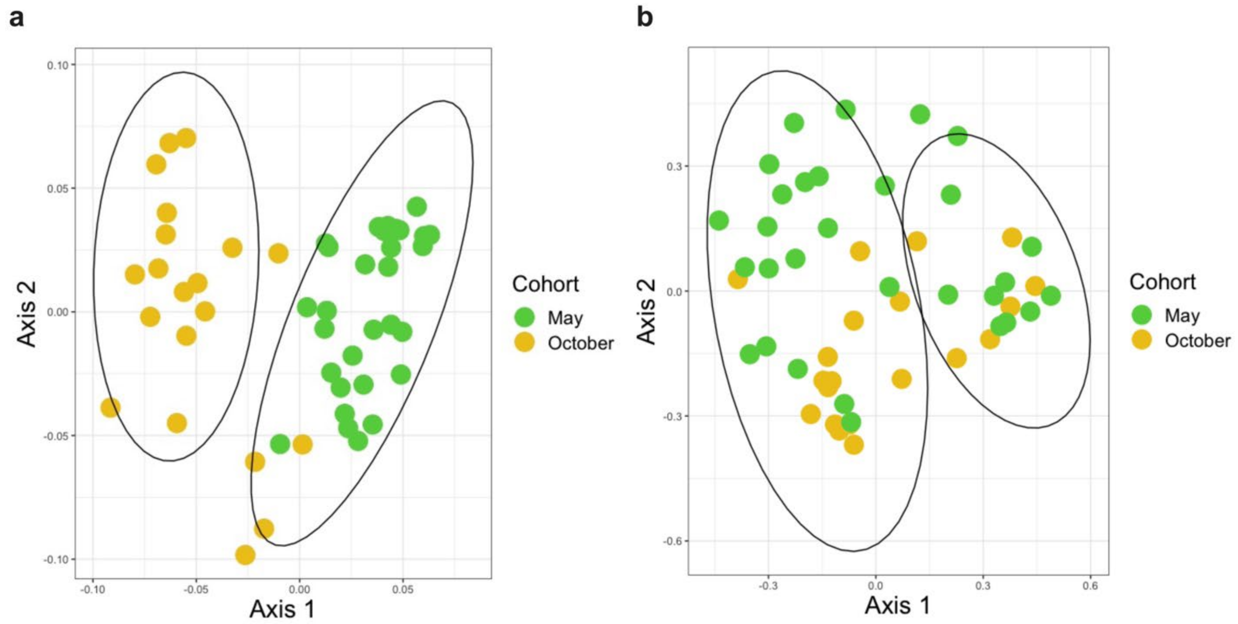

Supplemental Figure 2. Baseline intestinal microbial community composition differences between two cohorts (October, N = 20 and May, N = 30) of captive Jamaican fruit bats. **a.** Principal coordinate analysis showing weighted UniFrac distance by cohort. Ellipses denote significant (kmeans, $p < 0.05$) clusters. Points colored by cohort. **b.** Principal coordinate analysis showing Bray-Curtis distance by cohort. Ellipses denote significant (kmeans, $p < 0.05$) clusters. Points colored by cohort.

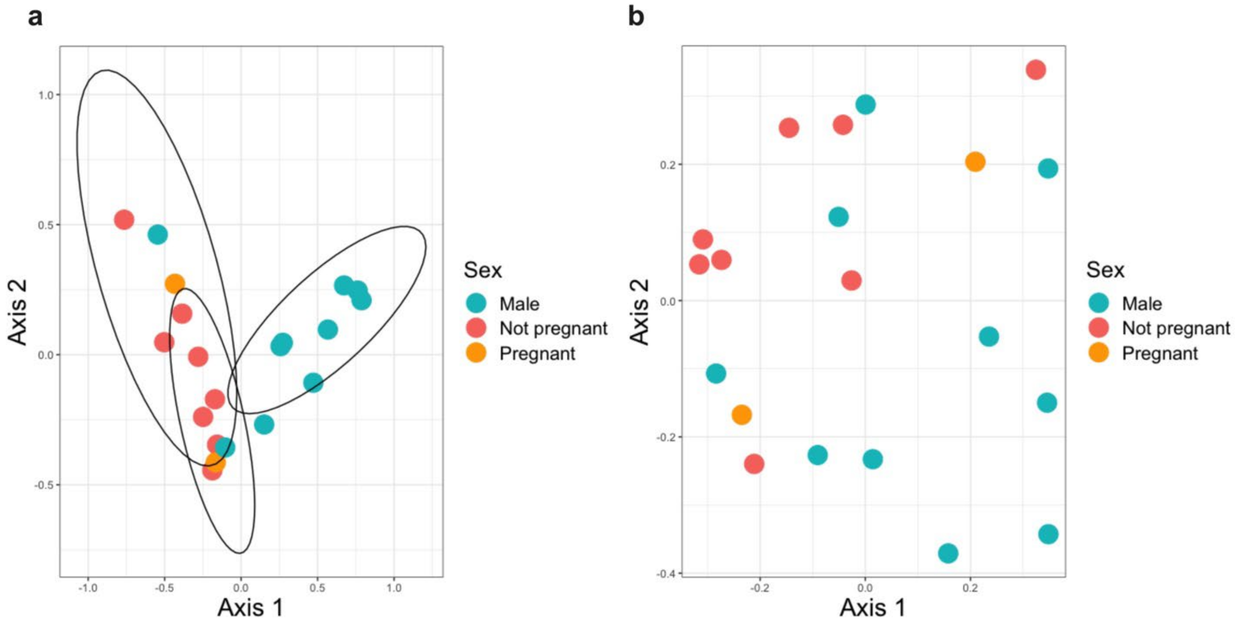

Supplemental Figure 3. Intestinal microbial community composition differences between male (N = 10) and female (N = 10) October cohort Jamaican fruit bats. **a.** Principal coordinate analysis showing weighted UniFrac distance by sex and pregnancy status. Ellipses denote significant (kmeans, $p < 0.05$) clusters. Points colored by sex and pregnancy status. **b.** Principal coordinate analysis showing Bray-Curtis distance by sex and pregnancy status. Lack of ellipses indicates lack of significant (kmeans, $p < 0.05$) clusters. Points colored by sex and pregnancy status.

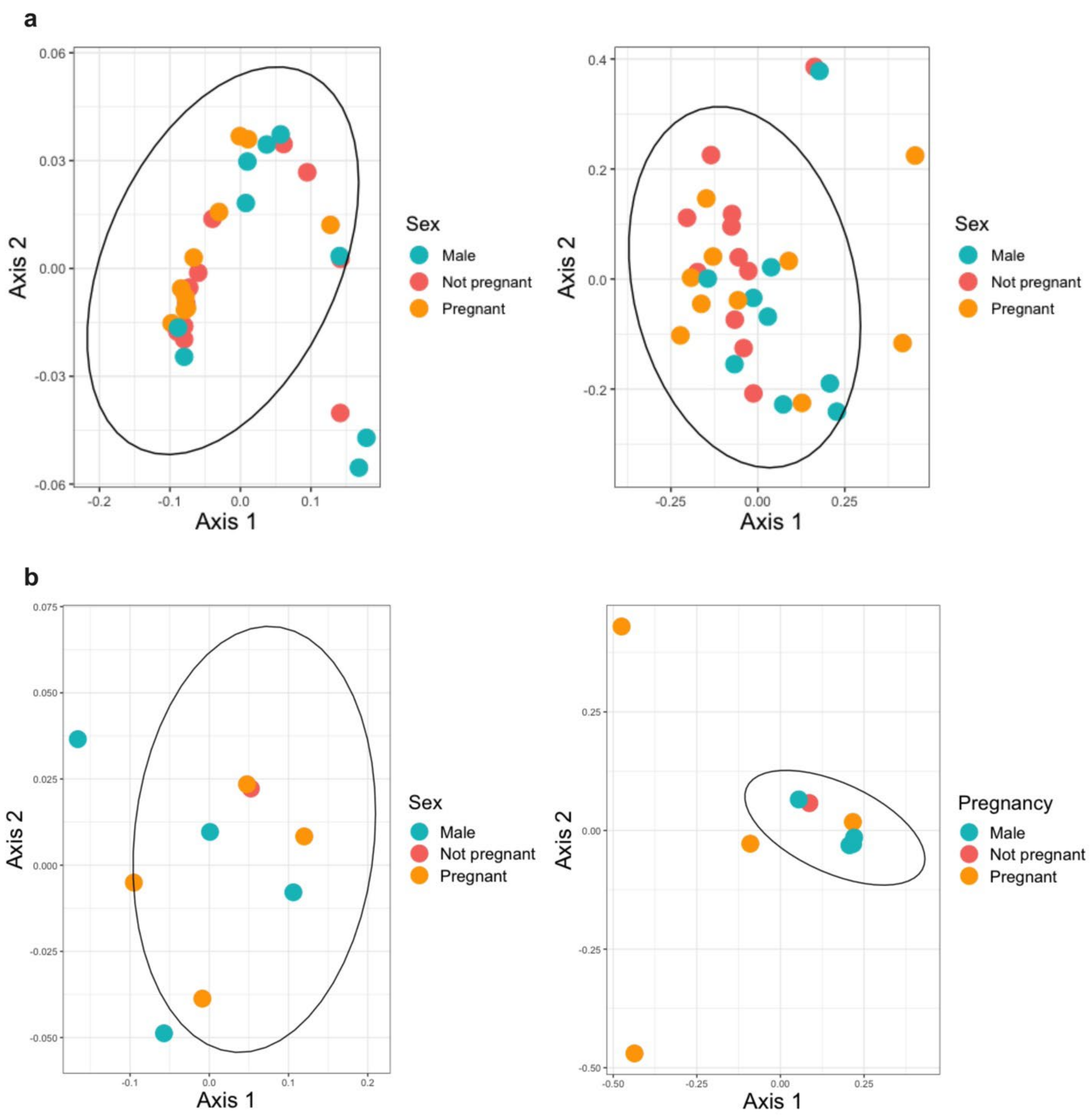

Supplemental Figure 4. Intestinal microbial community composition by sex in May cohort Jamaican fruit bats. **a.** Principal coordinate analysis showing weighted (left) and unweighted (right) UniFrac distance at D0 by sex (male, N = 9 and female, N = 21) and pregnancy status (pregnant, N = 10 and not pregnant, N = 11). Ellipses denote significant (kmeans, $p < 0.05$) clusters. Points colored by sex and pregnancy status. **b.** Principal coordinate analysis showing weighted (left) and unweighted (right) UniFrac distance at D28 by sex (female, N = 5 and male, N = 4) and pregnancy status (pregnant, N = 4 and not pregnant, N = 1). Ellipses denote significant (kmeans, $p < 0.05$) clusters. Points colored by sex and pregnancy status.

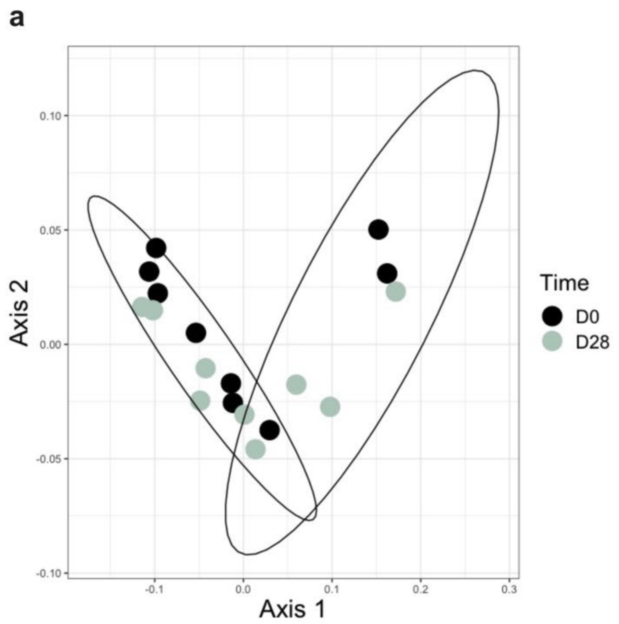

Supplemental Figure 5. Intestinal microbial community composition differences at baseline (D0, N = 9) and endpoint (D28, N = 9) in May cohort bats. **a.** Principal coordinate analysis showing weighted UniFrac distance by time. Ellipses denote significant (kmeans, $p < 0.05$) clusters. Points colored by time. **b.** Principal coordinate analysis showing Bray-Curtis distance by time. Ellipses denote significant (kmeans, $p < 0.05$) clusters. Points colored by time.

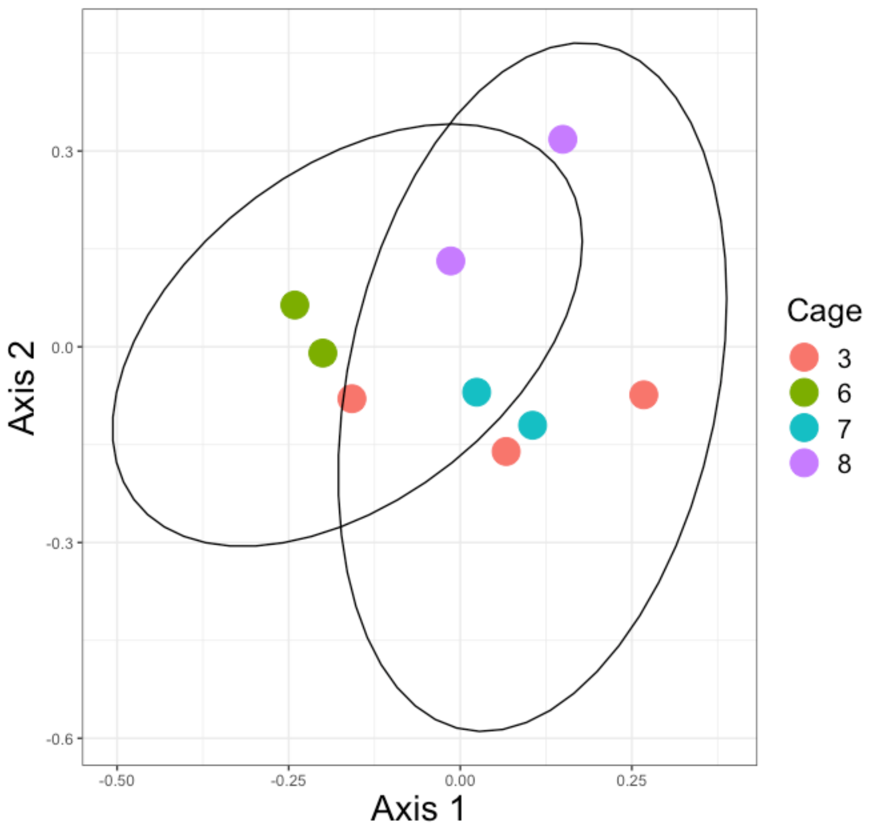

Supplemental Figure 6. No difference in endpoint microbial community composition by cage. Principal coordinate analysis of Bray-Curtis distance at D28 (N = 9). Points colored by cage. Ellipses denote significant (kmeans, $p < 0.05$) clusters.

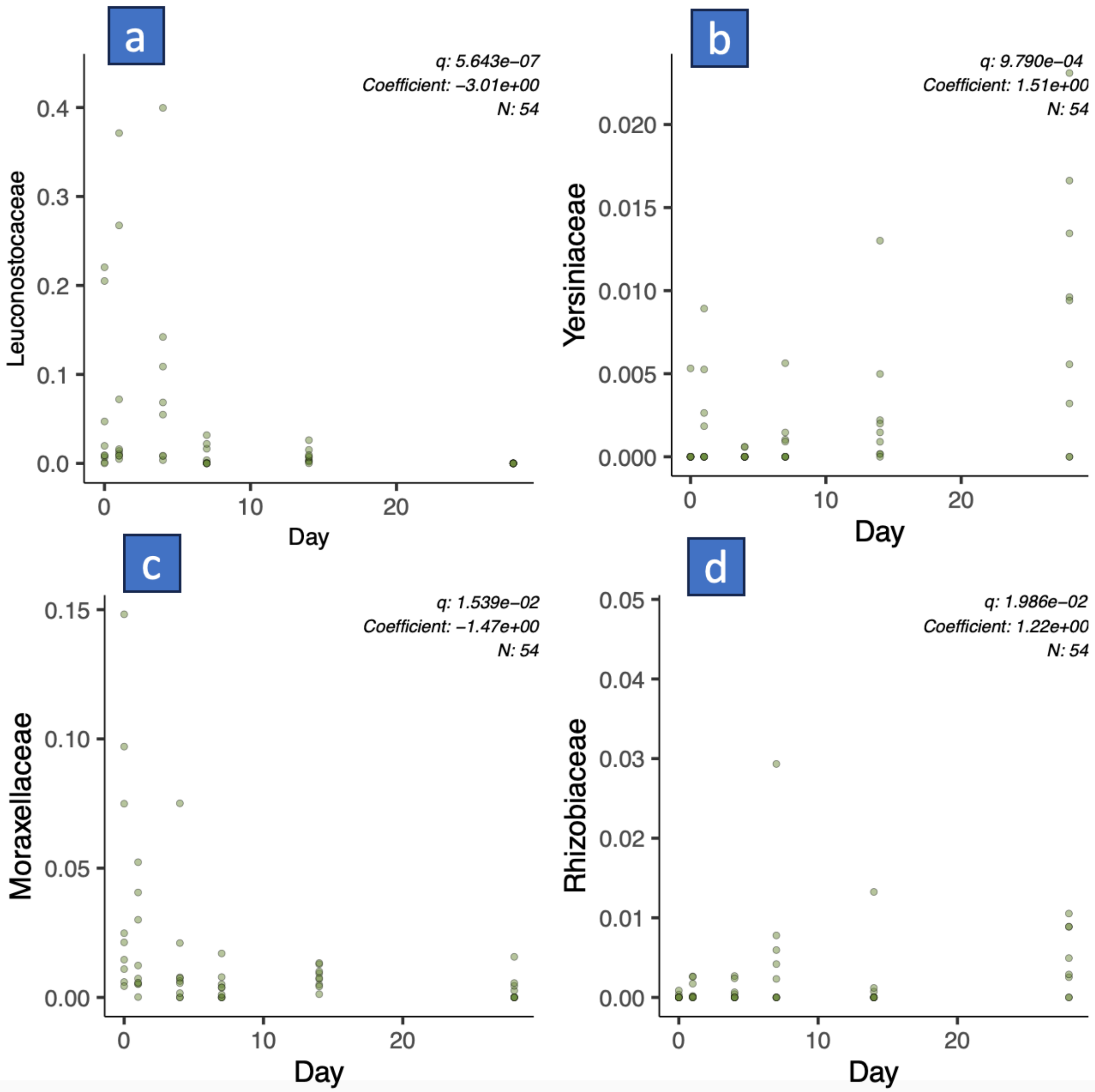

Supplemental Figure 7. Scatterplots showing relative abundance of four significantly different families over time in each bat in the May cohort. Benjamini-Hochberg corrected p-values, Maaslin2 coefficient, and sample size (N = 54) shown in top right corner. **a.** *Leuconostocaceae.* **b.** *Yersiniaceae.* **c.** *Moraxellaceae.* **d.** *Rhizobiaceae*.

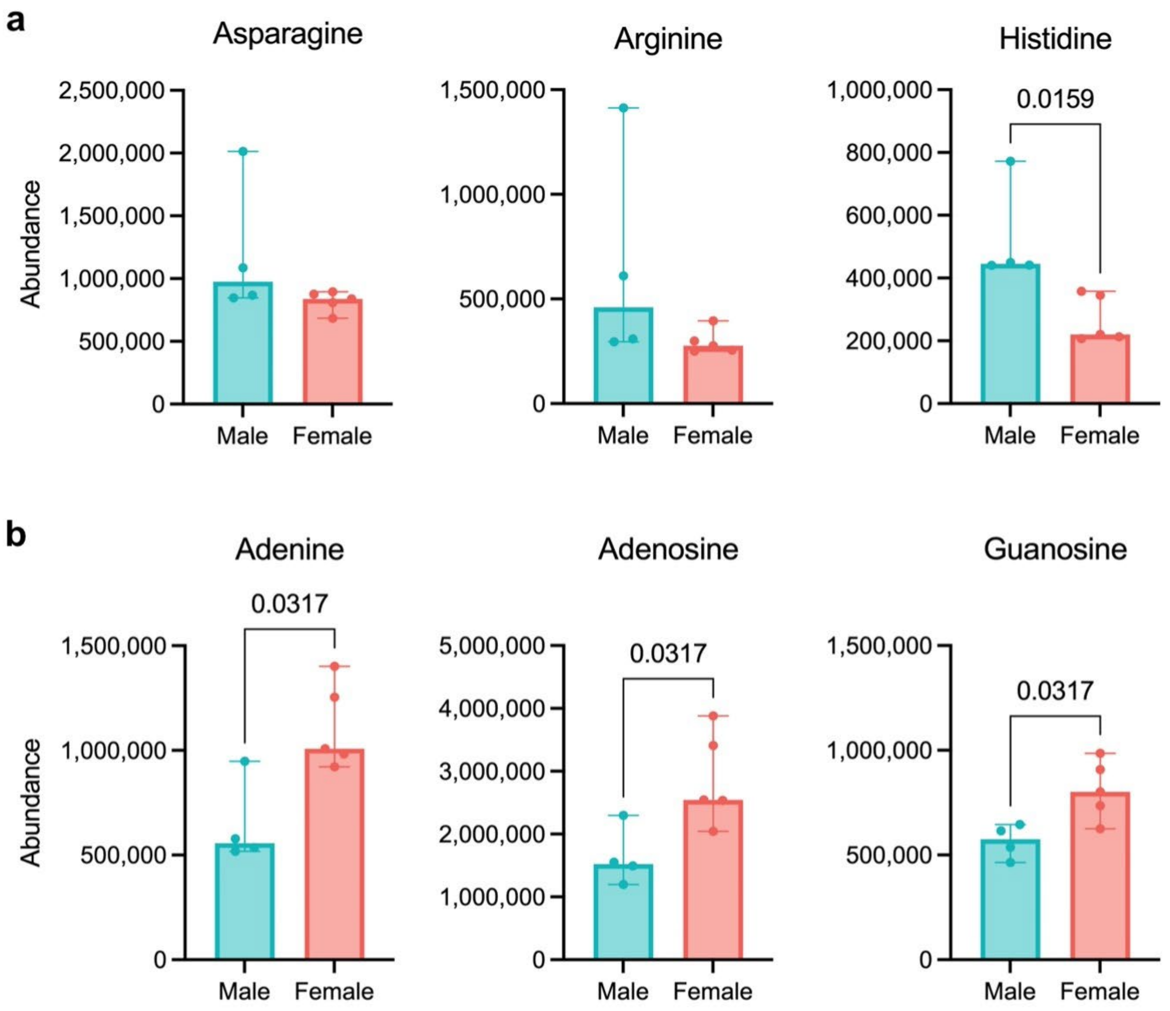

Supplemental Figure 8. Analysis of individual intestinal metabolites, as identified in sPLSDA analysis, in the Jamaican fruit bat May cohort by sex. **a.** Metabolites that were relatively more abundant in male bats than in female bats. Median and 95% confidence intervals with individual points overlaid. Significant p-values indicated. Mann-Whitney test of bats euthanized at D28 (N = 9). **b.** Metabolites that were relatively more abundant in female bats than in male bats. Median and 95% confidence intervals with individual points overlaid. Significant p-values indicated. Mann-Whitney test of bats euthanized at D28 (N = 9).

Purpose	Metric	Description	Formula
Microbial diversity	Simpson's diversity	Probability of two sampled features chosen at random being different	$1-\sum_{i=1}^{R} p_i^2$ p_i = proportion of observations belonging to species number i
	Shannon entropy	Estimate of both richness and evenness, with increased weight on richness	$-\sum_{i=1}^{R} p_i ln p_i$ p_i = proportion of observations belonging to species number i
	Faith's PD	Sum of branch lengths between observed features on a phylogenetic tree	$simple\ sum\ of\ branch\ lengths$
	Observed features	Simple count of features (sequence variants) observed in each sample	$simple\ sum\ of\ unique\ features$
Community composition	Weighted UniFrac	Proportion of branch lengths between microbes that are unique to samples, accounting for abundances	$\sum_{i}^{n} b_i \times \left\lvert \frac{A_i}{A_T} - \frac{B_i}{B_T} \right\rvert$ b_i = length of branch i A_i = abundances from branch i in sample A A_T = total number of sequences in sample A
	Unweighted UniFrac	Proportion of branch lengths between microbes that are unique to samples	$\frac{sum\ of\ unshared\ branch\ lengths}{sum\ of\ shared\ branch\ lengths}$
	Bray-Curtis	Calculation of the abundances of microbes shared between samples in relation to total numbers of microbes in each sample	$1-\frac{2C_{ij}}{S_i+S_j}$ C_{ij} = sum of the lesser abundances of species shared between samples i and j S_i = total number of microbes in sample i

Supplemental Table S1. Description and categorization of metrics used to calculate microbial diversity and community composition of Jamaican fruit bat rectal swabs.

Animal ID	Cohort	Sex	Pregnant	Sampled after acclimation	Sampled longitudinally
**1**	October	Female	No	Yes	No
**2**	October	Female	No	Yes	No
**3**	October	Female	No	Yes	No
**4**	October	Female	No	Yes	No
**5**	October	Female	No	Yes	No
**6**	October	Female	No	Yes	No
**7**	October	Female	No	Yes	No
**8**	October	Female	Yes	Yes	No
**9**	October	Female	Yes	Yes	No
**10**	October	Female	No	Yes	No
**11**	October	Male	N/A	Yes	No
**12**	October	Male	N/A	Yes	No
**13**	October	Male	N/A	Yes	No
**14**	October	Male	N/A	Yes	No
**15**	October	Male	N/A	Yes	No
**16**	October	Male	N/A	Yes	No
**17**	October	Male	N/A	Yes	No
**18**	October	Male	N/A	Yes	No
**19**	October	Male	N/A	Yes	No
**20**	October	Male	N/A	Yes	No
**21**	May	Female	No	Yes	No
**22**	May	Female	No	Yes	No
**23**	May	Female	No	Yes	No
**24**	May	Female	No	Yes	No
**25**	May	Female	Yes	Yes	No
**26**	May	Female	No	Yes	No
**27**	May	Female	Yes	Yes	No
**28**	May	Female	No	Yes	No
**29**	May	Female	Yes	Yes	No
**30**	May	Female	Yes	Yes	No
**31**	May	Female	No	Yes	No
**32**	May	Female	No	Yes	Yes
**33**	May	Female	Yes	Yes	Yes
**34**	May	Female	Yes	Yes	Yes
**35**	May	Female	Yes	Yes	Yes
**36**	May	Female	No	Yes	No
**37**	May	Male	N/A	Yes	No
**38**	May	Female	No	Yes	No
**39**	May	Female	No	Yes	No
**40**	May	Male	N/A	Yes	No
**41**	May	Female	Yes	Yes	No
**42**	May	Male	N/A	Yes	No
**43**	May	Female	Yes	Yes	Yes
**44**	May	Female	Yes	Yes	No
**45**	May	Male	N/A	Yes	No
**46**	May	Male	N/A	Yes	No
**47**	May	Male	N/A	Yes	Yes
**48**	May	Male	N/A	Yes	Yes
**49**	May	Male	N/A	Yes	Yes
**50**	May	Male	N/A	Yes	Yes

Supplemental Table S2. Animals included intestinal microbiome and metabolome analysis. Cohort, sex, pregnancy status, and sampling information are provided.
